# GRU-Based Denoising Autoencoder for Detection and Clustering of Unknown Single and Concurrent Faults during System Integration Testing of Automotive Software Systems

**DOI:** 10.3390/s23146606

**Published:** 2023-07-22

**Authors:** Mohammad Abboush, Christoph Knieke, Andreas Rausch

**Affiliations:** Institute for Software and Systems Engineering, Technische Universität Clausthal, 38678 Clausthal-Zellerfeld, Germany; christoph.knieke@tu-clausthal.de (C.K.); andreas.rausch@tu-clausthal.de (A.R.)

**Keywords:** automotive software systems development, real-time validation, hardware-in-the-loop (HIL), system integration testing, fault detection and clustering, deep learning, GRU-based denoising autoencoder, k-means

## Abstract

Recently, remarkable successes have been achieved in the quality assurance of automotive software systems (ASSs) through the utilization of real-time hardware-in-the-loop (HIL) simulation. Based on the HIL platform, safe, flexible and reliable realistic simulation during the system development process can be enabled. However, notwithstanding the test automation capability, large amounts of recordings data are generated as a result of HIL test executions. Expert knowledge-based approaches to analyze the generated recordings, with the aim of detecting and identifying the faults, are costly in terms of time, effort and difficulty. Therefore, in this study, a novel deep learning-based methodology is proposed so that the faults of automotive sensor signals can be efficiently and automatically detected and identified without human intervention. Concretely, a hybrid GRU-based denoising autoencoder (GRU-based DAE) model with the k-means algorithm is developed for the fault-detection and clustering problem in sequential data. By doing so, based on the real-time historical data, not only individual faults but also unknown simultaneous faults under noisy conditions can be accurately detected and clustered. The applicability and advantages of the proposed method for the HIL testing process are demonstrated by two automotive case studies. To be specific, a high-fidelity gasoline engine and vehicle dynamic system along with an entire vehicle model are considered to verify the performance of the proposed model. The superiority of the proposed architecture compared to other autoencoder variants is presented in the results in terms of reconstruction error under several noise levels. The validation results indicate that the proposed model can perform high detection and clustering accuracy of unknown faults compared to stand-alone techniques.

## 1. Introduction

During the development of modern automotive software systems (ASSs), the dependability attributes, i.e., reliability and safety, should be rigorously and comprehensively validated. In this way, the requirements of the automotive functional safety standard ISO 26262 [1] can be fulfilled. However, considering the increasing complexity of modern software-based vehicle systems with a high degree of functional dependencies [2], great attention should be paid to the development of advanced verification and validation methodologies.

Validating the target system at different stages of the development process can enable the detection and mitigation of unexpected faults. The fault or anomaly in the nonlinear multivariate systems is known as a deviation of one or more system parameters from normal behavior [3]. In ASSs, various locations are vulnerable to the occurrence of faults. Sensors, actuators, the communication network, ECUs, gateways, the power supply, and the data-logging system are the main potential points of faults in the vehicle [4]. Depending on the environmental and interior conditions, the duration of malfunction occurrence at the exact location varies, presenting as permanent, transient, or intermittent faults [5]. On the other hand, based on the characteristics of the sensed signals data, the faults can be classified into gain, offset/bias, noise, hard-over, spike, stuck-at, packet loss, delay, and drift faults [6]. According to V-model system development approach, different test phases are defined, namely model-in-the-loop (MIL), software-in-the-loop (SIL), processor-in-the-loop (PIL), hardware-in-the-loop (HIL), vehicle-in-the-loop (VIL) and real test drives [7].

To overcome the limitations of pure non-real-time simulation testing and real test drives on public roads, HIL simulation platform has been introduced as a safe, flexible, and reliable validation method [8]. In addition, HIL can benefit from the two aforementioned methods by conducting simulated test drives considering critical scenarios and real-time constraints [9]. However, due to the large amount of recorded data during test execution, conventional methods for analyzing the recordings that depend on experts with a deep understanding of the domain are not effective [4]. Consequently, the need for innovative solutions that reduce the cost, time and effort of the recordings analysis process has increased. By developing automated fault detection and diagnosis (FDD), potential risks and vulnerabilities can be effectively and accurately identified and eliminated at an early stage of system development. Along with ensuring the safety and reliability characteristics of the developed complex system, the cost and effort of unnecessary maintenance can also be reduced.

The literature on FDD scheme development shows a variety of approaches. The model-based [10], signal-based [11], knowledge-based [12], and data-driven approaches [13] are the main categories of FDD methods. Each strategy has its pros and cons. For example, in the model-based approach, despite its ability to identify the changes in the dynamic system state, the design of an accurate mathematical model of the complex system is considered a complicating factor [14]. Similarly, the knowledge-based approach, known as the rule-based approach, requires specific qualitative knowledge dictated by experts and extensive human intervention [15]. Moreover, for complex and large systems, the approach suffers from the inability to detect unknown faults that are not included in the historical data [16]. A signal-based approach is able to detect faults quickly and very effectively by analyzing the fault symptoms in the recorded signals without system modeling. However, the main drawbacks of this strategy are the difficulty in detecting and identifying unforeseen faults. Moreover, detecting similar symptoms in the presence of concurrent faults is still a difficult task.

Finally, the data-driven approach has proved its superiority in FDD by extracting the underlying structure and hidden patterns from the raw historical data without domain knowledge [17]. In recent years, great effort has been made to investigate data-driven approach, i.e., machine learning (ML) methods. The reason behind this is the rapid development of sensor technologies and the availability of sufficient computational resources. Nevertheless, the problem of availability of faulty datasets and the large amount and complexity of recorded data with redundant information requires more investigation [18]. To solve the problem of the manual extraction of representative features in ML methods, deep learning (DL)-based methods have been intensively researched in recent years. Thanks to the ability of DL methods to automatically extract and learn deep features from the dataset, the shortcomings of the traditional technique can be overcome.

In the state-of-the-art approach, several DL architectures have been proposed to tackle various application problems in different domains [19,20]. Among them, AE, GAN, CNN, DBN, and RNN have been widely investigated. Depending on the task, the methods are divided into anomaly/fault detection, fault classification, fault clustering, and fault regression. For example, in [21,22], several variants of AE were used as an unsupervised approach for detecting anomalies in the in-vehicle controller area network (CAN). On the other hand, to identify the type of faults and the faulty component of the system, several frameworks were proposed using the variants of RNN, i.e., GRU and LSTM, as the classification problem [23,24]. CNN techniques with one and two dimensions were employed for fault diagnosis in different vehicle systems, e.g., vehicle dampers in [25] and vehicle engines in [26]. Fault clustering is concerned with discovering the hidden relationship between the unlabeled data and grouping the data samples into multiple clusters. To accomplish this task, ML algorithms, e.g., k-means, are usually used with DL methods as proposed in [27]. Once the faults are classified, it is of interest to determine the current and future states of the components, which is known as fault prediction. For this purpose, LSTM was applied in [28,29] for battery fault prediction in electric vehicles as remaining-useful-life (RUL) prediction.

Despite the observable performance of the current intelligent approaches for FDD tasks, the developed FDD model still has various aspects to be investigated and explored. The main limitations can be summarized as follows: (1) In the developed FDD model, the focus is on a single fault without considering the occurrence of simultaneous faults. However, in a complex system, a combination of multiple independent faults may occur simultaneously at different locations and lead to multi-point failures [30]. (2) Under the real-world operating conditions of the complex system, the dataset is collected under non-realistic conditions, i.e., noisy and unbalanced data [31,32]. Most of the conducted studies on FDD models development, however, are developed using noise-free experimental data under normal conditions. (3) Only the known fault patterns in the target system are considered to address the FDD problem, which in turn leads to developing a model that cannot detect unforeseen faults or misidentifies the detected faults [33].

To bridge the mentioned gaps, in this study, a novel intelligent method for unknown fault detection and clustering (FDC) during the development process of ASSs is proposed. The developed model is capable of detecting and grouping unknown faults in the sequential data of sensor signals. To the best of our knowledge, the proposed method is applied for the first time to the detection and grouping of concurrent unknown faults during integration testing with real-time HIL simulation. The main contributions of the proposed study are summarized as follows:

- Based on deep features extraction and multi-level clustering, a novel methodology is developed using hybrid GRU-based denoising autoencoder (GRU-based DAE) and k-means to effectively address the problem of detecting and clustering single and simultaneous faults in the time-series data.
- Combining the proposed techniques enables detecting unknown faults under different noise levels, thus being appropriate for working under real industrial operating conditions with high robustness against noise.
- Furthermore, the comparison of different variants of the DAE architecture is analyzed and discussed using healthy and faulty validation data.
- Finally, the applicability and robustness of the proposed method are demonstrated using a high-fidelity simulation model of a complex gasoline engine with the entire vehicle dynamic system. On top of that, the dataset is collected under normal and faulty conditions using real-time fault injection considering the real-time constraints.

The rest of the article is organized as follows: The related work is presented in Section 2 highlighting the gabs and the main limitations. Following that, Section 3 introduces the proposed method. Afterward, as a case study, the target system setup and model training are illustrated in Section 4. Based on the real-time dataset from two automotive case studies, the evaluation results of the proposed model are discussed and presented in Section 5. Finally, conclusions and future research directions are outlined in Section 6.

## 2. Related Work

Nowadays, in manufacturing and industrial applications, the ML-based FDD approach plays a vital role not only in improving reliability but also in ensuring the safety of the developed systems. However, the availability of labeled data and unseen faults are the main challenges of the development process of the FDD model.

Towards overcoming the problem of lack of labeled data, unsupervised DL methods have motivated researchers to explore the possibilities of features learning from unlabeled data. Among them, AE has been introduced as a powerful technique not only to reduce the dimensionality of data but also to extract and learn the features from unlabeled data [34]. As a result, several research works have been conducted to investigate the applicability of AE for FDD in various applications based on historical time-series data [35]. Mallak et al. [36], for example, proposed a novel network architecture based on a hybrid LSTM-based autoencoder and a 1D CNN. The study focused on unsupervised and supervised learning methods so that the unprecedented sensor and component faults of hydraulic systems can be accurately detected and classified. The experimental results stated that the use of Pearson autocorrelation can surpass conventional methods in calculating the signal difference, which in turn leads to high detection performance. In the study, a balanced dataset from hydraulic test rigs was considered with three different fault types, i.e., stuck-at, gain, and offset. Concerning the same area, Wang et al. [37] performed a behavioral analysis of two DL-based FDD methods, i.e., stand-alone CNN and AE-based DNN with SoftMax classifier. The study investigated the best DL architecture that provides high classification accuracy with less computation time without data preprocessing. A simulation model of an MMC HVDC transmission network under seven conditions was employed as a case study to develop and validate the FDD model. The experimental results illustrate that the performance of the AE-based DNN is better than that of CNN in terms of accuracy, while the CNN requires less training and testing time. To improve the validation process of ASSs, a hybrid DL approach was proposed in [38] to automatically detect and classify individual sensor faults at the system level. To this end, hybrid CNN and LSTM were used to take advantage of each technique in extracting and learning the features required for FDC. In the mentioned study, the real-time constraints were considered in generating the healthy and faulty dataset by using a real-time fault injection framework. The superiority of the model in terms of detection and classification was demonstrated using a high-fidelity entire vehicle model as a case study.

To address the challenge of unknown faults, Han et al. [39] proposed an intelligent diagnosis model for rotating machinery based on the out-of-distribution (OOD) detection-assisted trustworthy approach. The novelty of this work is the development of an ensemble of five individual deep-base learners with trustworthy analysis so that the reliability and safety of the model can be ensured against unknown faults. By doing so, during the decision-making process, uncertainties can be identified, avoiding untrustworthy diagnoses in practical applications. To validate the proposed approach, wind turbine and gearbox systems are considered as two case studies. In the same context, to ensure the reliable operation of permanent magnet synchronous motors (PMSMs) under variable operating conditions, a novel robust CNN-based diagnosis model was proposed in [40]. The core idea behind the research is to consider the fault-related information in the transformed motor current signals for 2D instantaneous current residual images. Along with the rapid development of autonomous vehicles and ADAS, much attention has been paid to the utilization of ML techniques for the development of the FDD of such systems. In the automotive domain, in [41], the historical test recordings of real test drives were used to develop a robust ML-based model capable of detecting known and unknown faults under different driving scenarios. Towards this end, an ensemble ML classifiers was used so that the robustness of the model against data variability under different types of faults could be achieved. To validate the proposed methodology, recordings of road tests with injected faults during driving were used.

Besides the individual faults, one of the main complicating factors in the FDD process is the simultaneous occurrence of multiple single faults [42]. Additionally, in multivariate systems, acquiring data containing representative simultaneous faults with many possible combinations remains a challenge [43]. Therefore, in the last decade, considerable attention has been paid in various fields to developing FDD strategies for addressing this challenge. In this regard, Zhong et al. in [44] proposed an intelligent simultaneous FDD framework of vehicle engines based on a probabilistic committee machine (PCM). In this study, a real automotive case study, i.e., a real vehicle engine, was utilized to train and validate the proposed strategy. Depending on three real engine signals, 10 types of single faults and 4 reasonable combinations of simultaneous faults were explored. Considering the noisy operation process, a total of 2000 and 800 samples of single and simultaneous faults, respectively, was used. Compared to single probabilistic classifiers, the proposed method exhibited remarkable achievements in terms of FDD performance. Nevertheless, the accuracy can be improved by investigating the applicability of other techniques on the same real dataset. Similarly, in the field of building management systems, i.e., HVAC systems, great efforts have been made to develop methods and frameworks for concurrent FDD. For example, the results obtained by Wu et al. in [45] state that hybrid classification chains based on multiple labels with random forest can be effectively used to detect and classify single and simultaneous faults with 99.5% test accuracy. Specifically, in the study, focusing on actuator component, AHU was considered as a subsystem of the HVAC with six single faults and seven simultaneous faults. Although the proposed method achieved above-average performance compared to other methods, there is still room for improvement in terms of sensor FDD under noisy conditions and time constraints. Referring to the same problem in a solid oxide fuel cell system, Zang et al. argued in [46] that the problem of the multi-class classification of concurrent faults can be addressed using a multi-label DL network, i.e., a stacked sparse autoencoder. The novelty of the proposed method lies in the fact that independent and concurrent fault samples are not required. The features are automatically extracted from normal and unknown state samples of the target system. However, despite the demonstrated superiority of the proposed model for independent and concurrent faults, the study is limited to four possible combinations of the concurrent faults using non-real-time simulation data. Finally, one interesting approach towards the simultaneous FDD problem was proposed in [47] using the integration of GAN and continuous wavelet transform. The idea of the proposed study centers on the conversion of the sequential data into 2D time–frequency images, which, in turn, are used to construct the target model based on an adversarial learning mechanism. Thanks to the semi-supervised approach, a small number of labeled samples were used to overcome the challenge of availability of representative faulty data. Moreover, a real dataset from a gearbox test platform was used to analyze the effect of the training data on development, i.e., the number of labeled samples and the image size. The performance of the proposed system was evaluated under 15 different conditions and compared with that of other related works. The results demonstrated the improvement of the existing FDD methods in the literature in terms of classification accuracy with 99.16% on unlabeled data. An overview of the related work is presented in Table 1.

Judging from the above studies, current research in simultaneous FDD for ASSs development has not been sufficiently conducted. Unlike our proposed work, the current simultaneous FDD models have not considered either the real-time conditions or the problem of noise in the training dataset. Moreover, the fault classes are limited to certain conditions related to the target system. To address the aforementioned gaps, this study develops a novel hybrid DL-based methodology capable of detecting and clustering both single and concurrent faults during the development of ASSs, i.e., in the system integration phase using real-time HIL simulation.

## 3. Methodology

### 3.1. DL-Based Simultaneous Fault-Detection and Clustering Method

**Figure 1:**
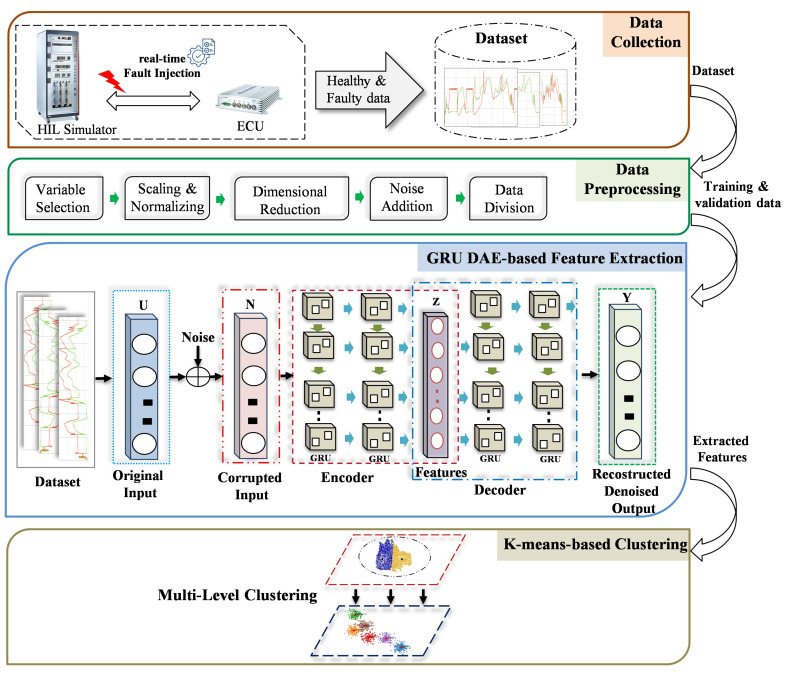
Proposed method for detection and clustering single and simultaneous faults.

In this section, the key stages of the development of the proposed FDC based on unsupervised DL techniques are outlined. The main advantage of the proposed method is the applicability to tackle the detection and clustering problem in the presence of noisy and unbalanced time-series data. By doing so, the real industrial scenarios can be simulated, where the faults are unknown as a deviation from the normal system behavior. Detection and clustering sensor signals’ faults of the ASSs during the HIL simulation-based testing is the target of the proposed model. This allows the analysis process of the resulting failures during HIL tests to be optimized in an efficient way by reducing the required time and effort. Developing the proposed model is accomplished through four phases, i.e., data collection, data preprocessing, features extraction, and detection and clustering phases as shown in Figure 1.

#### 3.1.1. Data Collection

**Figure 2:**
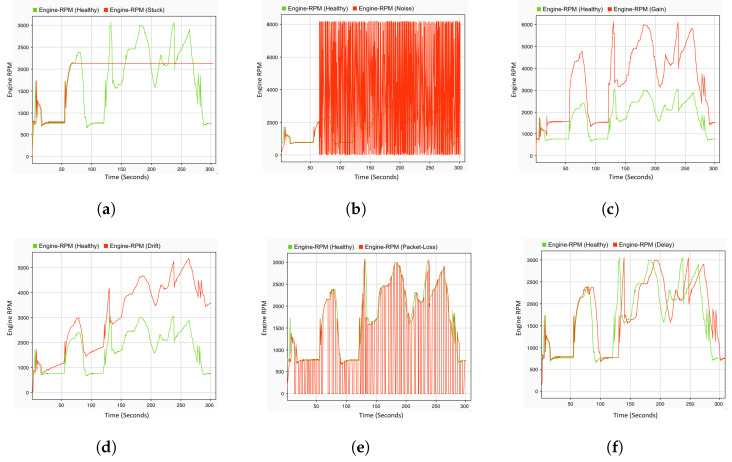
Fault types. (**a**) Stuck-at fault; (**b**) noise fault; (**c**) gain fault; (**d**) drift fault; (**e**) packet loss fault; and (**f**) delay fault.

To accurately capture the actual behavior of the target system under different types of faults, a real-time HIL simulation system is employed. Through this platform, the entire vehicle system can be simulated with high accuracy. Moreover, the interaction between the system under test, i.e., the ECU, and the controlled system can be accurately ensured in real time. By doing so, the data samples of the normal system behavior, i.e., the driving scenario, can be collected as multivariate time-series data. To generate representative fault data, the real-time fault injection developed in the previous study [48] was used. Various types of single and simultaneous faults are programmatically injected into the CAN bus in real time without changing the original system models. Some examples of the injected sensor faults are gain, stuck-at, drift, noise, delay, and packet loss. To collect the data of simultaneous faults, two combinations of the aforementioned fault types are injected in real time and simultaneously at two different locations. Both the single and concurrent faults data are saved as CSV files and forwarded to the pre-processing phase. Some examples of the selected fault types, compared to the healthy, are illustrated Figure 2.

#### 3.1.2. Data Preprocessing

Despite the fact that the data were generated in the context of a real-time simulation, additional preprocessing steps are required before training the model. The reason behind this is the need for the mitigation of irrelevant and unusable features avoiding negative effects on the training process [49]. By doing so, not only can the computational costs of the training be reduced, but also the problem of overfitting can be avoided. The main steps in this phase are variable selection, scaling and normalization, dimensional reduction, noise addition and data splitting. As ASSs have a high degree of complexity, a large number of sensor signals with high dependencies are available. In this study, specific sensor signals of the target system were selected so that the most relevant features are included in the model training. For this purpose, at the system level, the engine speed, engine torque, vehicle speed, throttle position, engine temperature, intake manifold pressure and rail pressure were selected as input variables for the detection and clustering. To enhance the performance of the training process, after variables selection, the feature is normalized and scaled using the Z-score normalization function. Thus, the range of the amplitude of the input feature is uniformly rescaled to the range [0–1]. A mathematical representation of the representative equation of the scaling step is given in [50]. Principal component analysis (PCA) [51] is used for dimension reductions. By doing so, the redundant information of the extracted features can be reduced, and representative features can be selected. To be specific, the correlated variables are transformed into independent variables, reserving the main variation factor from several variable vectors. Once the features are normalized and the dimensions reduced, the different noise levels are added according to the Gaussian distribution [52]. Specifically, random sample values from a Gaussian distribution with a mean of zero and a certain standard deviation are added to the original data. Finally, the preprocessed data are divided into three parts. The first part with 80% of the data is used for training the target model, while the second and third parts with 10% each are used for the validation and testing process.

#### 3.1.3. GRU-DAE-Based Feature Extraction

Due to the environment and complex operation conditions, most real-world automotive datasets contain uncertainty data with noise. This issue is not considered in the current DL-based FDD models under experimental conditions. This, in turn, leads to a negative effect on the accuracy of the developed model in real industrial applications. As a solution of the noisy measurements problem, recently, the DAE technique [53] exhibited remarkable achievements in enhancing the robustness of the fault-classification model against noise. As can be seen in Figure 1, it consists of four different parts, namely the input layer, corrupted layer, hidden layers, and output layer. The hidden layers are constructed into two main components, an encoder and a decoder [54]. After corrupting the original input data (U) by noise, the encoder focuses on converting the high-dimensional noisy input data (N) into a low-dimensional representation, often referred to as the latent code (Z). Mathematically, the encoder module of the GRU-based DAE is represented by Equation (1):

(1) $$Z=f_{enc}\left(WN+b\right)$$

where $f_{enc}$ represents an activation function of the encoder, *W* is an encoder’s weight, and *b* is an offset vector.

On the other hand, the function of the decoder is to map the latent code as output (*Y*) back into the original data space, i.e., to convert the extracted features into the original input. Thereby, a compact representation of the input data can be trained, which can be applied for dimensionality reduction, data denoising or generative modeling. Equation (2) represents the decoder module of the proposed architecture:

(2) $$Y=f_{dec}\left(W^{\prime }Z+b^{\prime }\right)$$

where $f_{dec}$ denotes an activation function of the decoder, and $W^{\prime }$ and $b^{\prime }$ represent the decoder’s weight and offset vector of the decoder, respectively. Thanks to the DAE structure, the noise and distortion can be removed from the input data by the encoding and decoding processes as de-noised reconstructed output data. However, to this end, the input data should be provided with a certain level of noise, e.g., Gaussian noise or corruption, so that the autoencoder can learn to denoise the data. In other words, the structure should be fed with two types of input data, i.e., noisy and clean data. The core idea behind the technique is to train the architecture by minimizing the reconstruction loss between the clean input data and the reconstructed denoised output. Mathematically, the loss function *L*(*U*, *Y*) is calculated according to Equation (3):

(3) $$L\left(U,Y\right)=||Y-{U||}^{2}$$

Notably, DAE can be developed based on different architectures depending on the type of data to be processed and the application domain [55]. Among them, AE based on GRU outperforms other AE architecture in detecting anomalies with multiple sensors, having lower complexity and inference time [56]. In our proposed architecture, GRU-based DAE is developed to provide the comprehensive extraction of representative healthy and faulty features considering noisy data. In the aforementioned variant, the advantages of GRU cells are leveraged in exploring the relationships and dependencies between multidimensional sequential data. Thus, multiple GRU layers of recurrent units series form both the encoder and decoder parts.

**Figure 3:**
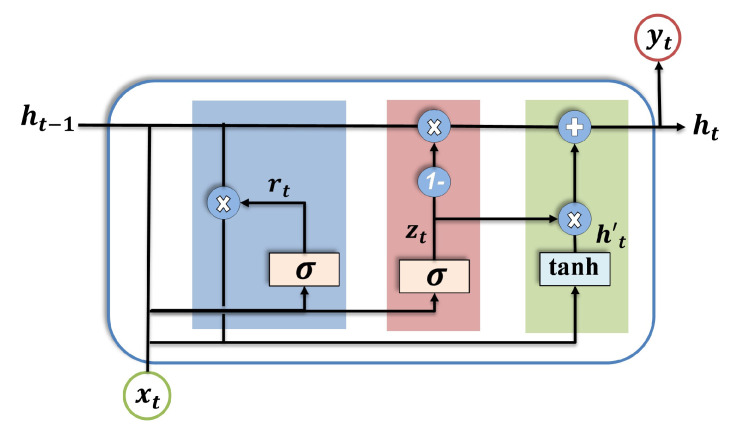
Internal structure of GRU cell.

Thanks to the structure of the LSTM, the limitation of the RNN, i.e., vanishing and exploding gradients, can be overcome. However, a complex architecture of LSTM requires a high overhead. Therefore, GRU was introduced to meet the requirements of classification tasks in real-time applications in terms of low resources and fast inference time [57]. The main innovation of GRUs is the use of gating mechanisms to control the flow of information, which makes them computationally more efficient and easier to train with fewer parameters compared to other types of RNNs. Due to the aforementioned ability of the GRU to capture long dependency in the data with a less complex architecture [58], it is selected in our study to construct the DAE model. The structure consists of a cell that stores information and two gate units for each block, i.e., the reset gate and the update gate. The function of the reset gate is to determine how much of the previous hidden state should be forgotten, while the update gate determines how much of the new information should be used to update the hidden state. The hidden state in a GRU summarizes the information from all previous time steps and serves as the input for the next time step. Figure 3 illustrates the GRU unit.

Mathematically, the output of the GRU cell can be described as follows:

(4) $$z_{t}=\sigma \left(W_{z}x_{t}+V_{z}h_{t-1}+b_{z}\right)$$

(5) $$r_{t}=\sigma \left(W_{r}x_{t}+V_{r}h_{t-1}+b_{r}\right)$$

(6) $$h_{t}^{\prime }=tanh\left(W_{h}x_{t}+V_{h}\left(r_{t}\times h_{t-1}\right)+b_{h}\right)$$

(7) $$h_{t}=\left(1-z_{t}\right)\times h_{t-1}+z_{t}\times h_{t}^{\prime }$$

where $z_{t}$ represents the GRU update gate; $x_{t}$ represents the input vector, i.e., noisy input; $h_{t}$ is the output vector, i.e., hidden state at time step *t*; *W* and *V* denote the weight; *b* represents the bias matrices; and (σ, tanh) are the gate and output activation function, respectively.

Table 2 describes the detailed specifications of the used GRU-based DAE architecture. In the proposed architecture, two GRU layers are used to form the encoder module, including 128 and 64 units, respectively. Furthermore, the batch normalization layer is established between the GRU layers. As a bottleneck layer in the encoder module, a dense layer is used. On the other side, similar to the encoder module, the mentioned layers are set symmetrically such that the first GRU layer contains 64 units and the second one 128 units.

As a result of training the GRU-based DAE with the backpropagation algorithm, the representative features of the healthy and faulty behavior can be extracted. The extracted features, i.e., the non-corrupted low-dimensional features, are then used in the clustering phase for detection and clustering tasks.

#### 3.1.4. K-Means-Based Multi-Level Clustering

Once the representative features are extracted by GRU-DAE in the previous phase, the denoised features are grouped into clusters through several levels using the k-means method [59]. The significance of the algorithm is that the grouping of the data samples can be performed as unsupervised learning without the need for the availability of labeled data. Thus, the hidden patterns can be extracted from the data without any knowledge of the true labels. k-means clustering has achieved remarkable success in addressing various technical challenges, such as social tags, shape recognition and wireless sensor networks [60]. The algorithm’s main principle is founded on the idea of categorizing the features into several groups according to their similarity. By doing so, the data points with similar characteristics will belong to the same cluster with the closest mean value. In other words, the distance between the data samples and each cluster center is calculated. Then, the objective of the training is to minimize the sum of squared distances within the clusters so that the samples can be assigned to the clusters that are closest to each center. Mathematically, the sum of squared distances can be represented in [61].

In this study, the objective is to tackle the problem of the detection and clustering of unknown faults.Therefore, the labels of fault classes, i.e., ground truths, are unavailable. To achieve high performance of detection and clustering, multi-level feature clustering is implemented, i.e., three levels for single fault and two levels for concurrent faults. At the first level, the fault detection task is carried out by separating the healthy and faulty features. Based on the grouped faulty features, in the case of a single fault, the faulty components can be determined by separating the corresponding faulty features, e.g., faulty features of accelerator pedal position (APP) and RPM sensor. Finally, the last level is performed to group the features of a specific type of faults, e.g., gain or delay, in the case of single and concurrent faults.

Two main phases are performed to find the optimal cluster centroids and achieve convergence, i.e., cluster assignment and centroid update. In the first phase, the Euclidean distance between the data points and each centroid is calculated. In the second phase, each centroid is updated based on the calculation of the mean of all data points assigned to a cluster. Note that the extracted features are not labeled, and the method should be able to cluster not only the samples of single faults but also the samples of simultaneous faults with different combinations. In this study, 5 clusters are selected to represent single faults and 10 clusters for their combinations. To avoid the drawback of k-means when processing massive amounts of data, the PCA technique is employed before clustering. By identifying the underlying structure in the data, the high-dimensional data are converted into low-dimensional data, which in turn reduces the computational effort and load.

## 4. Case Study and Experimental Implementation

This section presents the experimental setup and implementation steps of the proposed method. The structure of the target system, which is used as a case study to demonstrate the applicability, is described. To evaluate the performance and robustness of the proposed model, two different automotive case studies, namely a gasoline engine system and a dynamic vehicle system, are utilized.

### 4.1. HIL Real-Time Simulation System

**Figure 4:**
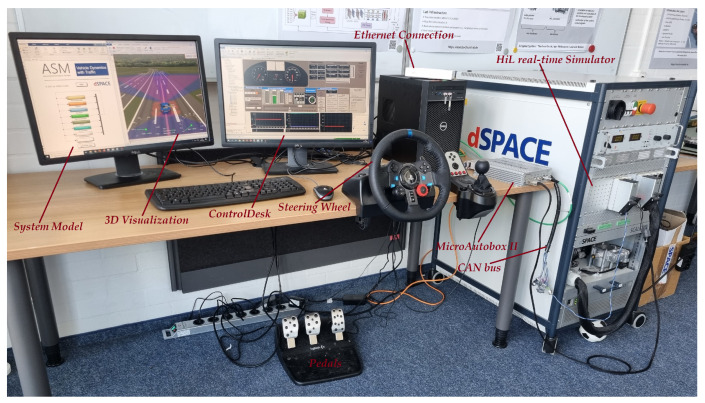
Real-time virtual test drives environment.

The ability of automatic code generation from a complex model using a model-based development approach has paved the way for significant advancements in the field of real-time simulation. The HIL system has been introduced as an effective tool for a safe, reliable and reproducible real-time verification and validation platform. Therefore, in this study, to simulate and execute the systems of selected case studies in real time, the HIL system is used. Figure 4 illustrates the major hardware components of the HIL system, i.e., HIL simulator (dSPACE SCALEXIO), MicroAutoBox as real ECU, and physical communication. The models are run in real time such that the ECU model is deployed and executed in MicroAutoBox, and the rest of the whole vehicle model is deployed and executed in SCALEXIO. Performing the experiments involves the use of four software tools, namely, ModelDesk, ConfigurationDesk, MotionDesk and ControlDesk [62].

### 4.2. Case Study 1: Gasoline Engine

**Figure 5:**
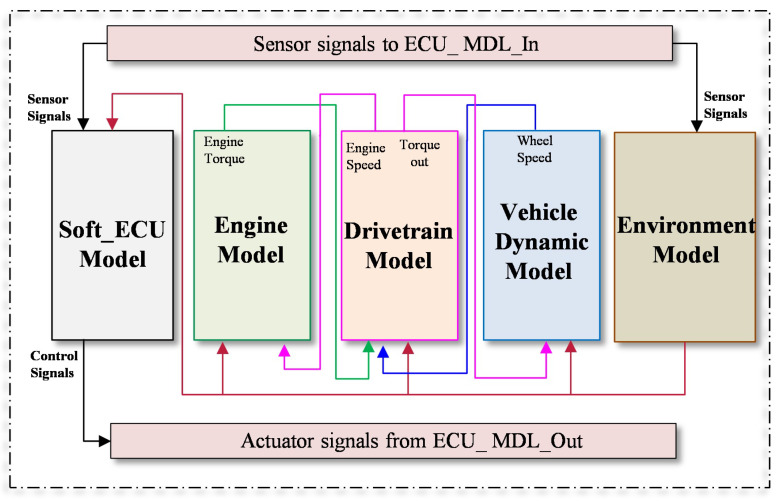
System architecture of the selected case study.

Due to its essential role in the vehicle system, and according to ISO 26262, the engine management system is classified as classes C to D of the Automotive Safety Integrity Level (ASIL). Therefore, faults in such a system should be rigorously analyzed and mitigated during the development process. In this study, a high-fidelity system model of the ASM gasoline engine provided by dSPACE [63] is used. The behavioral model is designed in the MATLAB/Simulink environment covering all subsystems and components so that the detailed characteristics of the system can be modeled comprehensively. To represent the interaction characteristics of the target system with the other vehicle subsystems, the powertrain model, vehicle dynamics model, and environment model are also considered as illustrated in Figure 5. To simulate the real ECU, the control algorithm of the target system is also modeled in the form of connected Simulink blocks. In this case study, the ECU model of the engine system represents the system under test (SUT). The connection between the SUT and the mentioned vehicle subsystems is realized by using the CAN bus protocol employing the Real-Time Interface CAN Multimessage Blockset (RTICANMM). The structure of the engine system consists also of several subsystems, i.e., air path system, fuel system, piston engine system, and exhaust system.

Since the modeled system provides high accessibility to the internal variables, the sensor and actuator components are selected for the potential fault location in this study, in particular, the crank angle sensor, battery voltage sensor, accelerator pedal sensor, ignition and starter demand, EGR mass flow, engine speed, intake and exhaust manifold pressure, fuel pressure, throttle, coolant temperature sensor and railbar sensor. Various types of sensor faults are considered for this purpose, i.e., gain, stuck-at, noise, delay, and packet loss. Notably, the aforementioned fault types are programmatically injected in real time via RTICANMM without changing the system model.

### 4.3. Case Study 2: Vehicle Dynamics with Traffic

**Figure 6:**
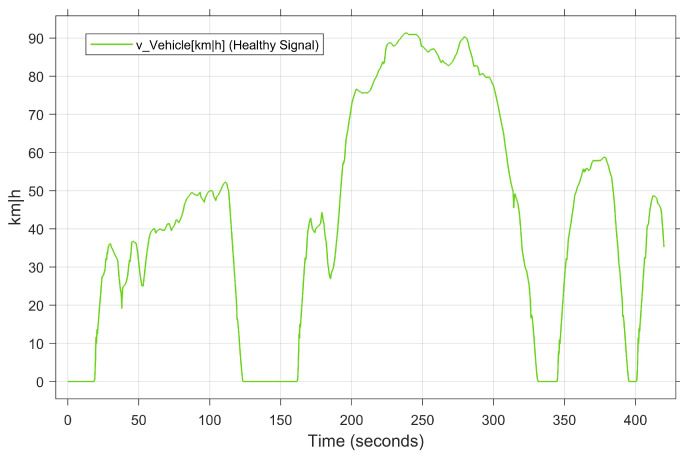
Driving scenario of the selected case study.

The objective of the second case study is to validate the performance of the developed model in a real driving scenario. However, to avoid the limitations of a real test drive in terms of time, cost per test kilometer, and risk to the test driver, this study employs a real-time simulated test drive. Specifically, the ASM vehicle dynamics model from dSPACE [63] is used to develop the real-time digital test drive platform in our laboratory. As shown in Figure 4, the platform includes a HIL simulator, MicroAutoBox II as a real ECU, CAN bus communication, driving elements, and a 3D driving environment model. The developed platform enables both manual and automatic driving modes. Thanks to dSPACE’s ModelDesk and MotionDesk development tools, the driving environment and roads can be designed and modeled precisely and flexibly. What results is that the validation data can be generated based on the user’s behavior in real time for our proposed DL-based FDC. The main steps of the execution are the setting of the internal and external system parameters, the design/selection of the driving environment, i.e., the road and driving conditions, the configuration of the recording data system, and the real-time executions. The driving scenario of conducting the digital test drive is illustrated in Figure 6 as a vehicle speed over the time.

### 4.4. Data Description

Once the test configurations are specified, the driving scenarios can be manually/ automatically executed. Under fault-free conditions, standard system behavior, including signals from the sensors and actuators, can be captured in real time, generating a healthy dataset. For case study 1, to conduct automatic test drives, the highway is selected from the list of driving scenarios as a predefined scenario (see Figure 6). In case study 2, on the other hand, to manually perform a user-based test drive, our developed real-time test environment is used. The time sampling rate of the recording system is set to 0.01 s. The throttle position [%], engine temperature [degC], mean effective engine torque [Nm], engine speed [rpm], intake manifold pressure [Pa], rail pressure [bar] and vehicle speed [Km|h] are selected in both case studies as input variables of the proposed model. To generate the faulty dataset, the aforementioned five fault types are injected into the target components, i.e., the APP sensor and engine speed sensor, individually and simultaneously. For this purpose, the real-time FI framework developed in the previous work is used. Detailed information about the possible fault types in the time-series data can be found in [64,65].

**Figure 7:**
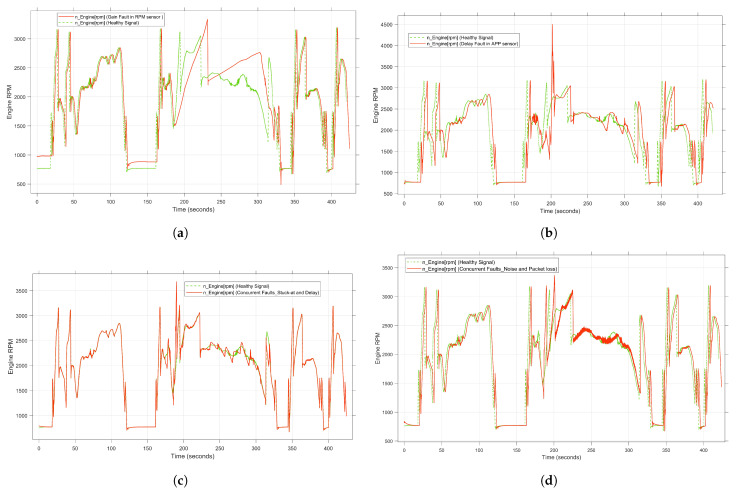
System behavior under single and concurrent transient faults. (**a**) Effect of gain fault as a single fault. (**b**) Effect of delay fault as a single fault. (**c**) Effect of stuck-at and delay fault as concurrent faults. (**d**) Effect of noise and packet loss fault as concurrent faults.

Focusing on generating a representative dataset with high coverage, the faults are injected permanently and transiently. In the case of transient faults, the faults are injected during real-time execution between 170 and 330 s, while the permanent faults are injected throughout the entire drive cycle. As a result, 856,000 samples of faulty data are generated, including five individual faults. In addition, concurrent fault samples are collected from ten different combinations of two fault types. To achieve this, two different faults are injected into the APP sensor and the speed sensor simultaneously. Figure 7 illustrates the individual and simultaneous faults generated by the fault injection method. Table 3 presents the generated data for each experiment corresponding to each type of fault and its combinations. The dataset is generated in the form of CSV files containing all the selected variables. The size of each CSV file is about 63,422 KB, which, in the sum of all files, gives a sufficient dataset for the DL approach with a size of 2.6 GB. Afterward, the collected data are passed to the preprocessing stage so that the data are cleaned, normalized, and split into three parts. In case study 1, for training the target model, 80% of the generated time-series data, i.e., healthy and faulty data, is randomly selected, whereas to optimize the decision threshold, 10% is used for validation. Additionally, 10% is used for the testing process. As a consequence, no labeling process is imposed on the dataset. On the other hand, all generated data from case study 2, i.e., 180,000 samples, are used to validate the developed model. To overcome the problem of robustness to noise, different noise levels are added to the dataset according to the Gaussian mechanism.

Some examples of the generated faulty data compared to the healthy data can be seen in Figure 7. Specifically, Figure 7a,b illustrate the system behavior in the presence of single fault, whereas the effect of the concurrent faults is presented in Figure 7c,d.

### 4.5. Training and Optimization of DAE

**Figure 8:**
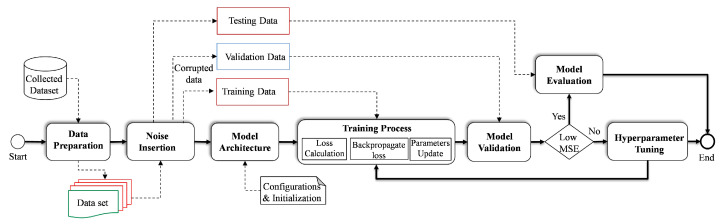
Flowchart of model training and optimization.

**Figure 9:**
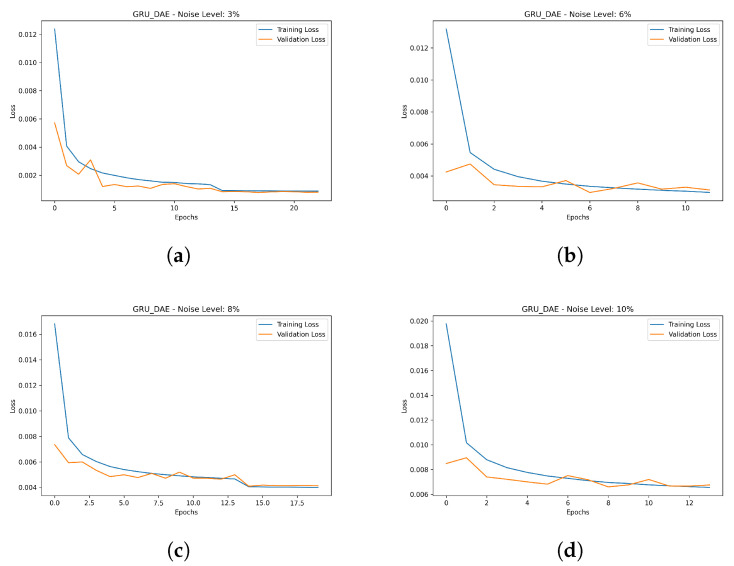
Hyperparameter optimization results with training and validation accuracy. (**a**) Under level noise 3%. (**b**) Under level noise 6%. (**c**) Under level noise 8%. (**d**) Under level noise 10%.

The initial step after capturing the data from the real-time simulation is data preparation, whereby the data are cleaned and reprocessed. Once the data are pre-processed, Gaussian noise is artificially added to the data depending on the desired noise level. Following the addition of the noise, the data are divided into three parts, i.e., training, validation and testing, as shown in Figure 8. Prior to the training process, the configurations of the model architecture are specified, i.e., the parameters and hyperparameters are initialized. It is noteworthy that the hyperparameters play a crucial role in the performance of the trained model. Therefore, in this study, to build an effective model, a range of hyperparameters are selected, covering the number of layers, number of GRU unit, learning rate, batch size, noise level, activation function and the optimizer. To optimize the target model, validation data samples are used so that the hyperparameter values are tuned according to the defined range. The criteria of selecting the hyperparameters is to reach convergence with high accuracy. Considering this strategy, different DAE variants, i.e., ANN-DAE, CNN-DAE and LSTM-CNN-DAE, are trained. The list of optimized hyperparameters of the proposed GRU-based DAE architecture is summarized in Table 4. To provide better comparison, the convergence curves of the proposed trained model as a result of the training process under different levels of noise, i.e., 3%, 6%, 8% and 10%, are illustrated in Figure 9a–d, respectively. Concerning the optimization phase, the tuning of the hyperparameters is one of the well-known challenges in the development of DL models. Hence, in this study, the technique of auto-tuning is used so that the hyperparameters can be dynamically adjusted during the optimization process. Specifically, after initializing the model parameters, the training process is monitored, i.e., the loss and performance metrics are tracked. Then, the target parameter is updated accordingly until convergence is achieved.

## 5. Results and Discussion

The testing and evaluation results of the proposed model are presented in this section, highlighting the superiority of the proposed architecture compared to other models. Furthermore, the ability of the proposed model to overcome the challenge of simultaneous faults under different noise conditions is demonstrated using the test data samples.

### 5.1. Evaluation Metrics

According to the proposed methodology, the results are discussed in two phases: feature extraction and clustering. For this purpose, the reconstruction error, i.e., the mean square error MSE, is used to evaluate GRU-DAE. On the other hand, to assess clustering performance, the Davies–Bouldin (DB) score is used.

The reconstruction error is an evaluation measure to determine how well the developed model is able to reproduce the data pattern after the coding and decoding process. The MSE [66] represents the loss function of the DAE design so that the reconstruction error between the original input and the reconstructed denoised data can be qualified. The lower the reconstruction error, the better the data representation of the trained model. The goal of training and optimizing the designed model is to minimize the reconstruction error so that the essential features can be accurately captured and reconstructed. The mathematical equation for the MSE is presented in Equation (8), where $X_{i}$ is the samples of the original input data, $X_{i}^{\prime }$ is the samples of the reconstructed output and *L* is the total number of data points:

(8) $$\mathrm{MSE}=\frac{1}{L}\Sigma {\left(X_{i}-X_{i}^{\prime }\right)}^{2}$$

The DB score is widely used for developing unsupervised clustering models as a quality assessment measure [67]. The idea behind this measure is to determine the degree of similarity between clusters and their respective centers. In other words, the ratio between the similarity within a cluster and the dissimilarity between clusters is used to calculate the average of the maximum similarity of each cluster set. A lower value indicates better clustering performance with precisely separated clusters. Thus, the search for the optimal number of clusters aims to minimize the ratio value, while taking into account the problem of overfitting. At the elbow point, where the inertia value increasingly changes, the optimal number of clusters is considered the best. Mathematically, the calculation of the DB score is given in Equation (9). *C* represents the number of clusters, *D* is the average distance between cluster data points and its centroid, and *L* is distance between the centroids of clusters $c_{i}$ and $c_{j}$:

(9) $$\mathrm{DB}=\frac{1}{C}\Sigma max\frac{\left(D_{i}-D_{j}\right)}{L(c_{i},c_{j})}$$

In addition to the above evaluation metrics for unlabeled data, several quantitative evaluation measures are used to assess the performance of the proposed model. In particular, based on predefined labeled testing data, the precision, recall and *F*1-score are calculated to evaluate the fault-detection and clustering performance of the developed architecture [68]. The percentage of the total classification results that were correctly predicted can be identified by precision, while recall represents the correctly identified elements as a percentage of all elements to be identified. Finally, to calculate the harmonic mean between precision and recall, the *F*1-score is used. Mathematically, the quantitative assessment metrics used can be represented by Equations (10)–(12), based on the true positive (TP), false positive (FP) and false negative (FN) values:

(10) $$Precision=\frac{TP}{TP+FP}$$

(11) $$Recall=\frac{TP}{TP+FN}$$

(12) $$F1-Score=2*\frac{Precision*Recall}{Precision+Recall}$$

### 5.2. GRU-DAE Performance Compared to Other AE Variants

In this study, the superiority of the proposed model is demonstrated by comparing its performance with other AE variants. For this purpose, ANN-AE, ANN-DAE and CLSTM-DAE are implemented. All developed models are validated using the same test samples. In Table 5, the high performance of the proposed model in extracting the noise-free features with a low MSE of 0.0073 is evident. Notably, ANN-DAE performs well under noise-free conditions with a low MSE value of 0.0212.

Besides the clean data, the applicability of the proposed model in denoising the data is illustrated at different noise levels compared to the other models. In this case, four different levels of Gaussian noise are added, i.e, 3%, 6%, 8% and 10%. Although the MSE value, in our proposed model, is increased with increasing noise, the MSE value at a high noise level is still acceptable compared to that of the other models, i.e., 0.0618 at noise 10%. On the other hand, CLSTM-DAE shows poor outcomes with a MSE value of 0.5641 due to its complex structure. Besides the simple structure, ANN-DAE also shows good performance at different noise levels, i.e., 0.0316, 0.0530, 0.0706 and 0.0766. However, compared to our proposed model, further improvement is needed to achieve higher accuracy.

### 5.3. Evaluation Results under Different Noise Levels and Fault Classes

**Figure 10:**
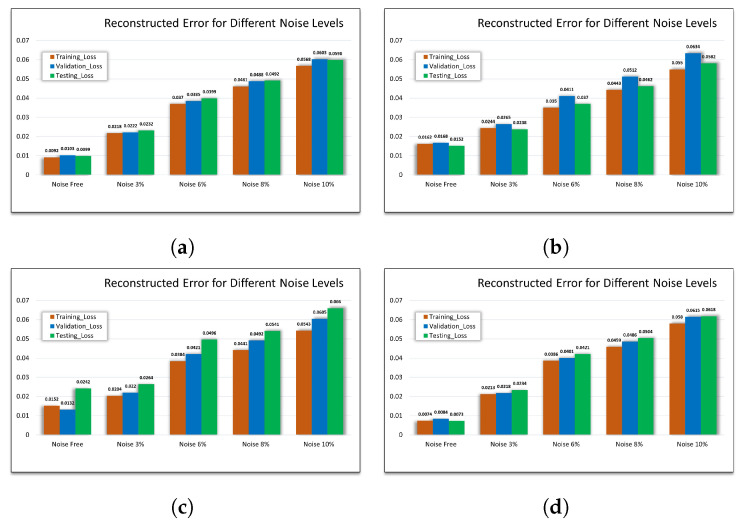
Effect of the data size and fault classes on the performance. (**a**) MSE using 25% of data with 3 fault classes. (**b**) MSE using 50% of data with 7 fault classes. (**c**) MSE using 75% of data with 11 fault classes. (**d**) MSE using 100% of data with all classes fault classes.

To demonstrate the effect of the data size and number of fault classes on model performance, the proposed model is trained under different conditions. In other words, the ability of the proposed model to extract and reconstruct the features under different data sizes and different number of fault classes is illustrated. As shown in Figure 10, 4 CSV, 8 CSV, 12 CSV and all CSV files are used to train the model under different levels of noise, i.e., noise-free, 3%, 6%, 8%, and 10%. The model exhibits high performance under a low number of fault classes with an MSE of 0.00992 under fault-free conditions. However, the higher the level of disturbance, the larger the MSE value, which reaches 0.0598 at 10% disturbance. On the other hand, the effect of the size of the dataset used, including the faulty sample, can be observed in the fact that the MSE value increases as the fault classes increase. The reason behind this is the increase in the diversity of the faulty features in the case of combination faults along with low data samples. Nevertheless, even with high noise and including all fault classes, the performance of the model is still reasonable with MSE of 0.0618 at 10% noise. Thus, the model performance in the presence of a high degree of fault diversity can be ensured by increasing the size of the training data.

### 5.4. Clustering Results of Single and Concurrent Faults

**Figure 11:**
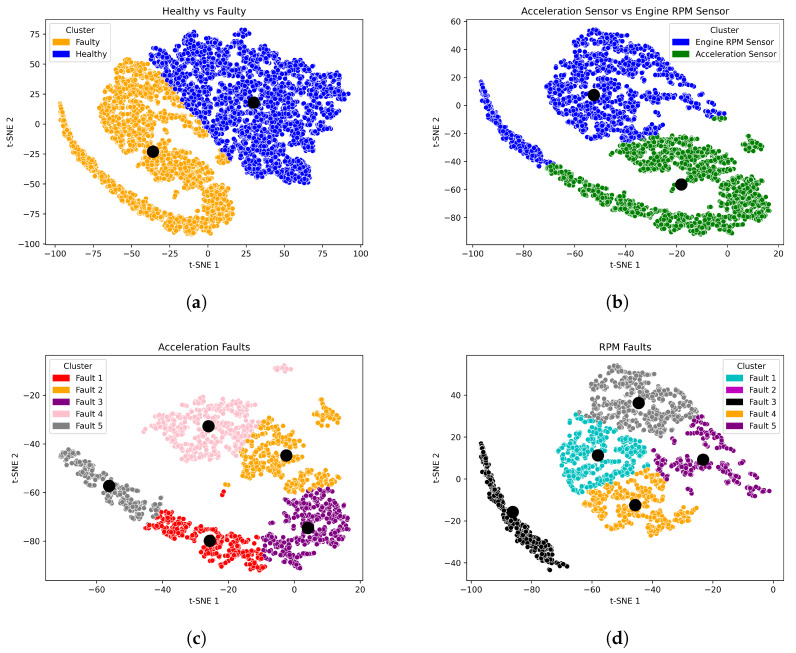
Feature visualization of the multi-level clustering for single fault including the clusters’ centres. (**a**) Level 1 clustering of the faulty features. (**b**) Level 2 clustering of the faulty components features. (**c**) Level 3 clustering of the fault types features. (**d**) Level 3 clustering of the fault types features.

**Figure 12:**
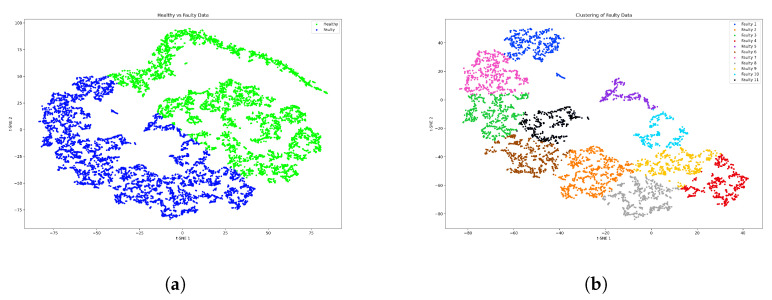
Feature visualization of the multi-level clustering for concurrent faults. (**a**) Level 1 clustering of the faulty features. (**b**) Level 2 clustering of the fault types features.

In this study, a multi-level clustering strategy is employed to accurately categorize the extracted features in the presence of single and simultaneous faults. To this end, three levels of clustering are performed so that the faulty features can be grouped without negatively affecting the healthy features. Specifically, in the case of a single fault, as shown in Figure 11, the extracted features are divided into two clusters at the first level, i.e., healthy and faulty. At the second clustering level, the faulty features are grouped according to the faulty components, either APP faults or RPM faults. Finally, at the third level, the features of APP faults and RPM faults are grouped into specific clusters representing the nature of each fault. However, in the case of a simultaneous fault, the characteristics of the faulty components cannot be precisely determined. Therefore, the cluster levels are limited to two levels. Similar to a single fault, the healthy and faulty features are separated in the first level. Subsequently, the faulty features are clustered into subgroups representing the fault classes that are combined to form the concurrent faults. Figure 12 illustrates the cluster levels of the extracted features in the case of concurrent faults.

**Figure 13:**
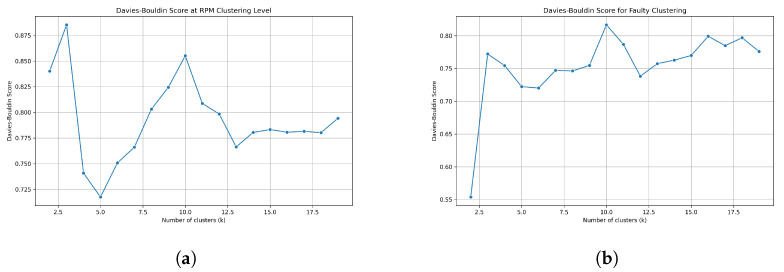
Optimization of number of clusters selection. (**a**) Clusters selection for single fault clustering. (**b**) Clusters selection for concurrent faults clustering.

To select the optimal number of clusters, the inertia value is applied. In this strategy, the inertia value is calculated against the number of clusters. Here, the objective is to select the appropriate number such that the inertia value is minimized. As a result, the optimal number is chosen at the points where the change in inertia value occurs increasingly, i.e., at the elbow point. The chosen number of clusters depending on the inertia value is shown in Figure 13a,b for single and simultaneous faults, respectively.

As a measure for evaluating the performance of the cluster model, the DB index in Table 6 and Table 7 shows how well the features are separated and clustered for single and concurrent faults, respectively. In the case of a single fault, the low value of DB indicates high performance at all cluster levels. At the first level, the DB score is 0.6442, while at the second level, it is 0.6875, increasing slightly due to the similarity of the features of the faulty components. The best score is obtained by clustering the faulty features of APP with a DB index of 0.5695.

In the case of concurrent faults and due to the increasing complexity of the extracted features, the DB score is high compared to the single fault case. More specifically, the DB score in the first stage is 0.7838, while in the second stage, a DB score of 0.7859 is achieved. Nevertheless, the above values indicate the high performance of clustering, even in the presence of simultaneous combination faults compared to conventional k-means.

In conclusion, by applying our proposed approach, robust representations against the noise of the input can be trained, enabling more accurate fault clustering and pattern identification, even in the presence of noise.

### 5.5. Fault-Detection and Clustering Results of Case Study 2

In the context of DL-based FDD development, it is essential to ensure that the developed model is applicable in real industrial application. Therefore, in this study, the dataset of system behavior under healthy and faulty conditions is recorded as testing data based on real-time manual driving. The recordings of the digital test drives in this case contain healthy, single and simultaneous faults. To be noted, due to the driver behavior and real-world conditions, the test data are considered uncertainty samples.

The test results prove the effectiveness and superiority of the proposed model in the reconstruction of the original denoised data with low loss, i.e., MSE of 0.0955. The MSE shows that the proposed model can process uncertain real datasets containing noise with a low reconstruction error. Moreover, a remarkable achievement is observed in the clustering phase by clustering the faulty data. In other words, using the proposed model, the faults can be accurately detected in single and concurrent occurrences with low DB score, i.e., 0.4872 and 0.5617, respectively. The low score of DB for single faults indicates that the detection performance is better than that of simultaneous faults. This is due to the high complexity of the representative features of simultaneous faults signal patterns. To evaluate the detection and clustering performance of the proposed method, the recall, precision and F1-score are considered the quantitative performance criteria. To this end, the aforementioned dataset of a real-time digital test drive containing the labels of healthy and faulty samples is used. In the case of a individual fault, a remarkably high detection performance is achieved with a precision of 99.17%, a recall of 95.23% and an F1-score of 97.16%. The results analysis shows that single faults can be accurately detected with a very good positive predictive value and a high true positive rate. Furthermore, in the case of concurrent faults, the high detection accuracy demonstrates the reliability of the proposed model, i.e., precision 92.83%, recall 98.09% and F1-score 95.38%. It is worth noting that in the case of compound faults, a number of test data samples are incorrectly clustered as faulty, i.e., falsely reported. The reason behind this issue is the similarity of data samples in some cases between the healthy and faulty samples. This problem can be further addressed by conducting experiments-based failure analysis to define the threshold for behavioral deviations that are indicative of faults. Remarkably, the mentioned accuracy is also affected by the degree of complexity of the fault patterns. In other words, some data samples of faults types are similar to each other, which in turn leads to incorrect diagnosis results.

## 6. Conclusions

In this article, a novel DL-based method is proposed to tackle the problem of detecting and clustering unknown sensor faults during the validation process of ASSs. The core of the developed method is the adoption of a GRU-based denoising autoencoder with the k-means algorithm. The denoised extracted features by our method contribute to a significant improvement in the detection and clustering process under noisy conditions. Thus, the reliability and robustness of the clustering model for single and simultaneous faults under different levels of noise can be ensured. Compared to other restructuring models, our proposed architecture exhibits high superiority in terms of reconstruction error under noisy conditions. To verify the effectiveness of the developed method, the real-time simulation data of two automotive case studies are used, i.e., gasoline engine and dynamic vehicle system with traffic. The experimental results show that the proposed model is not only able to extract representative features effectively but also to cluster the faults more accurately compared to stand-alone techniques. Specifically, based on the DB index, the results show high performance in the clustering process of single and simultaneous faults with scores of 0.4872 and 0.5617, respectively. Quantitative evaluation metrics are used to validate the diagnostic effectiveness of the model based on a testing dataset from a digital test drive. In both cases of fault occurrence, i.e., individually and simultaneously, the model achieves high detection and clustering performance with an average accuracies of 97.16% and 95.38%, respectively. Overall, the proposed model is able to detect the potential risks and vulnerabilities at an early stage of the system-development process. This, in turn, leads not only to improve the safety and reliability aspects but also to reduce the costs and effort of unnecessary maintenance.

As future work, the proposed framework can be further extended to cover the root-cause identification tasks, i.e., to identify the faulty components of the developed systems and its causes. To this end, based on the data-driven approach, historical data with representative faulty sensors and actuators to develop the target model are required. Therefore, novel techniques for intelligent fault detection and localization need to be explored, considering the problem of availability of representative faulty data.

## Figures and Tables

**Table 1:** Overview of the related work.

Reference	Techniques	Application Domain	Dataset	Robustness	Faults	Remarks
[36]	LSTM-based autoencoder	FDD for hydraulic systems	Hydraulic test rig dataset	Not considered	Permanent, single fault	Accuracy: low 71% Target: sensor and components faults. Real-time constraints: not considered.
[37]	AE-based DNN	FDI of modular multilevel converters systems	Simulation dataset	Not considered	Permanent, single fault	Accuracy: high 99.7% Target: current sensors. Real-time constraints: not considered.
[41]	Ensemble classifier-based ML	Known and unknown faults detection in automotive test recordings	Real test drive dataset	Considered	Transient, single fault	Accuracy: low 85% Target: sensors and connection signals. Real-time constraints: not considered.
[44]	PCM	Diagnosis simultaneous faults of automotive engine	Real engine dataset	Considered	Transient, single and simultaneous faults	Accuracy: low 88.74% validation: sensors signals. Real-time constraints: considered.
[45]	Classifier chains and random forest	Simultaneous FDD of air handling unit (AHU) system	Real AHU dataset	Not considered	Transient, single and simultaneous faults	Accuracy: high 99.5% Target: actuator signals. Real-time constraints: considered.
[46]	Stacked sparse autoencoder	Classifying the faults of solid oxide fuel cell (SOFC) systems	Simulation dataset	Not considered	Permanent, single and simultaneous faults	Accuracy: low 79.94% Target: actuator signals. Real-time constraints: not considered.
[47]	GANs with CWT	Intelligent Single and simultaneous FDD of the gearbox	Real gearbox test bench dataset	Considered	Permanent, single and simultaneous faults	Accuracy: high 99.16% Target: sensors signals. Real-time constraints: considered.
Proposed work	GRU-based DAE and k-means	Single and simultaneous FDC for HIL testing of ASSs	Real-time simulation dataset	Considered	Transient, single and simultaneous faults	Accuracy: 0.9538%, DB score 0.4872 Target: sensors signals. Real-time constraints: considered.

**Table 2:** The architecture parameters of the proposed model.

Layer	Output Shape	Parameters
input${}_{2}$ (InputLayer)	[(None, 4)]	0
reshape${}_{3}$ (Reshape)	(None, 1, 4)	0
gru${}_{4}$ (GRU)	(None, 1, 128)	51,456
batch normalization${}_{2}$ (BatchNormalization)	(None, 1, 128)	512
gru${}_{5}$ (GRU)	(None, 64)	37,248
dense${}_{2}$ (Dense)	(None, 4)	260
reshape${}_{4}$ (Reshape)	(None, 1, 4)	0
gru${}_{6}$ (GRU)	(None, 1, 64)	13,440
batch normalization${}_{3}$ (Batch Normalization)	(None, 1, 64)	256
gru${}_{7}$ (GRU)	(None, 1, 128)	74,496
dense${}_{3}$ (Dense)	(None, 1, 4)	516
reshape${}_{5}$ (Reshape)	(None, 4)	0
Total params: 178,184 Trainable params: 177,800 Non-trainable params: 384		

**Table 3:** Faults and collected dataset description.

Fault ID	Fault Type	Fault Duration	Training Samples	Testing Samples
H	Healthy	-	2,352,000	294,000
F1	Gain	165–320 s	2,352,000	294,000
F2	Stuck-at	172–330 s	2,352,000	294,000
F3	Noise	175–310 s	2,352,000	294,000
F4	Packet loss	170–312 s	2,352,000	294,000
F5	Delay	170–325 s	2,352,000	294,000
F1F2	Gain and Stuck-at	166–338 s	2,352,000	294,000
F1F3	Gain and Noise	168–340 s	2,352,000	294,000
F1F4	Gain and Packet loss	179–329 s	2,352,000	294,000
F1F5	Gain and Delay	177–334 s	2,352,000	294,000
F2F3	Stuck-at and Noise	174–330 s	2,352,000	294,000
F2F4	Stuck-at and Packet loss	180–327 s	2,352,000	294,000
F2F5	Stuck-at and Delay	173–324 s	2,352,000	294,000
F3F4	Noise and Packet loss	166–320 s	2,352,000	294,000
F3F5	Noise and Delay	179–328 s	2,352,000	294,000
F4F5	Packet loss and Delay	176–336 s	2,352,000	294,000

**Table 4:** Optimal Hyperparameters for GRU-DAE architecture.

Hyperparameter	Optimal Values
GRU Layers	4
Dense Layers	2
GRU Unit	384
Batch Normalization Layer	2
Epochs	850
Batch Size	128
Learning Rate	0.0001
Activation Function	adam
Optimizer	tanh
Noise Level	10%

**Table 5:** Reconstruction error of GRU-DAE compared to other AE variants.

Noise Level	ANN-DEA	CLSTM-DAE	Proposed GRU-DAE
Noise-free	0.0212	0.5518	0.0073
Noise 3%	0.0316	0.5551	0.0234
Noise 6%	0.0530	0.5617	0.0421
Noise 8%	0.0706	0.5633	0.0504
Noise 10%	0.0766	0.5641	0.0618

**Table 6:** Davies–Bouldin scores of the proposed model for single fault.

Clustering Level	GRU DAE + K-Means	Stand-Alone K-Means
Level 1	0.6442	1.2541
Level 2	0.6875	0.9560
Level 3	0.7170	0.9586
Level 3	0.5695	0.9312

**Table 7:** Davies–Bouldin scores of the proposed model for concurrent faults.

Clustering Level	GRU DAE + K-Means	Stand-Alone K-Means
Level 1	0.7838	2.0676
Level 2	0.7859	1.2288

## Data Availability

Data available on request due to restrictions.
